# Public health round-up

**DOI:** 10.2471/BLT.22.011022

**Published:** 2022-10-01

**Pakistan humanitarian emergency**

Two men, one carrying food supplies, wade through floodwaters surrounding the village of Malook Khaskhali in Matiari District, Sindh province, Pakistan. Starting in June, torrential monsoon rains have caused widespread flooding that has damaged villages and infrastructure in all four provinces of Pakistan. As of 3 September, an estimated 634 000 people had been obliged to move into camps, and 6.4 million were in urgent need of humanitarian aid.

**Figure:**
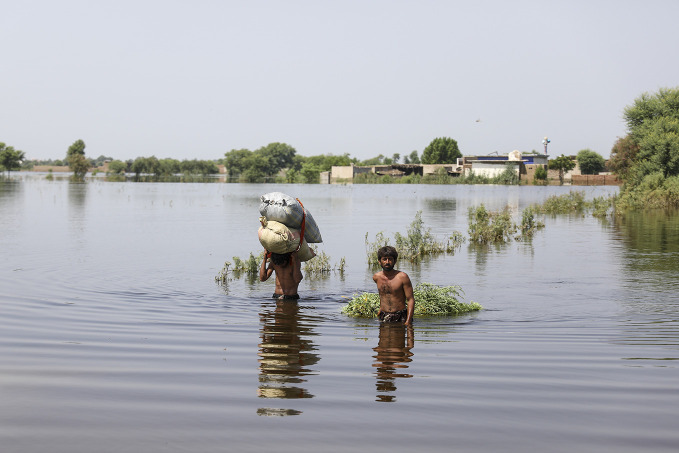
UNICEF/Asad Zaidi

## Pakistan emergency response

On 25 August, the government of Pakistan declared a state of emergency due to flooding resulting from severe monsoon rains and unusually heavy glacial runoff that followed a severe heat wave.

As of 9 September, over 33 million people had been impacted, including 634 000 who had been displaced and were living in camps. Some 6.4 million people were judged to be in urgent need of humanitarian aid. An estimated 12 500 had been injured and 1290 killed. Impacts on the health system included damage to over 1460 health facilities.

Early disease surveillance revealed an increase in cases of diarrhoea, malaria and typhoid. Dengue, measles, leishmaniasis, coronavirus disease 2019 (COVID-19) and polio were assessed to be at increased risk of transmission if the situation was not rapidly contained.

The World Health Organization (WHO) is supporting efforts to ensure that the affected population has access to the essential health services and products. On 9 September two shipments containing 15.6 metric tonnes of cholera kits, water and multipurpose tents were sent from the International Humanitarian City in Dubai, United Arab Emirates to Karachi, Pakistan. Mobile health teams were redirected to flood-affected areas, and more than 4500 medical camps were set up by the Pakistani government, WHO and health partners.

WHO has also scaled up disease surveillance in flood-affected areas and delivered essential diagnostics including over 230 000 rapid tests for cholera, malaria and dengue.

https://bit.ly/3xgRS4z

## New Ebola flare-up

Health authorities in the Democratic Republic of the Congo declared a resurgence of Ebola on 22 August, following the confirmation of a case in the town of Beni in North Kivu. A 46-year-old woman died of Ebola virus disease on 15 August 2022, having initially received care for other ailments at the Beni Referral Hospital.

The Beni and Goma branches of the country’s National Institute of Biomedical Research confirmed Ebola virus in samples taken from the patient. Analyses showed that the virus was genetically linked to the 2018–2020 outbreak in North Kivu and Ituri Provinces – the country’s largest, most protracted outbreak.

“Ebola resurgences are occurring with greater frequency in the Democratic Republic of the Congo,” said Matshidiso Moeti, WHO Regional Director for Africa. She noted however that the health authorities in North Kivu have successfully stopped several Ebola flare-ups and predicted that this outbreak would be brought under control quickly.

^https://bit.ly/3BgMrVT^

## Ebola treatment recommendations

WHO published its first guideline for Ebola virus disease therapeutics, which included strong recommendations for the use of two monoclonal antibodies to treat the disease.

Following a systematic review and meta-analysis of randomized clinical trials, WHO recommended that patients receive the neutralizing monoclonal antibodies, mAb114 (Ansuvimab; Ebanga) and REGN-EB3 (Inmazeb) as soon as possible after laboratory confirmation of diagnosis.

Published on 17 August, the new guidance complements clinical care guidance that outlines the care Ebola patients should receive to put them on the best path to recovery.

“Provision of best supportive medical care to patients, combined with monoclonal antibody treatment […] now leads to recovery for the vast majority of people,” said co-chair of the guideline development group, Robert Fowler, of the University of Toronto, Canada.

https://bit.ly/3RJt1OE

## Pandemic response fund established

A new fund dedicated to pandemic prevention, preparedness and response (PPR) was established. Launched by its governing board at their 8–9 September inaugural meeting, the PPR fund will provide a dedicated stream of additional, long-term financing to strengthen PPR capabilities in low- and middle-income countries and address critical gaps through investments and technical support at the national, regional and global levels.

The fund will draw on the comparative advantages of key institutions engaged in PPR, provide complementary support, improve coordination among partners, incentivize increased country investments, serve as a platform for advocacy, and help focus and sustain much-needed, high-level attention on strengthening health systems.

The first calls for proposals for investments by the PPR fund will open in November 2022.

https://bit.ly/3RCgZqR

## Global framework for life science use

WHO issued a global guidance framework to support responsible use of the life sciences. Launched on 13 September, the Framework calls on leaders and other stakeholders to mitigate biorisks and safely govern “dual use” research (research which has clear potential benefits but can also be misused to cause harm).

The first global, technical and normative framework of its kind, the Framework seeks to address challenges that include preventing the accidental and deliberate misuse of biology and other life sciences, as well as optimizing governance and oversight to both accelerate and spread innovation, while mitigating negative impacts.

"Life sciences and technologies offer many opportunities to improve our health, our societies and our environment” said Soumya Swaminathan, WHO Chief Scientist. “However, developments and advances in life sciences and associated technologies could pose risks caused by accidents during experiments, inadvertent and deliberate misuse.”

https://bit.ly/3Bz66R1

## Long COVID in Europe

At least 17 million individuals across the 53 Member States of the WHO European Region may have experienced post COVID-19 condition, or long COVID, in the first two years of the pandemic.

This is according to the results of a modelling exercise conducted for the WHO Regional Office for Europe by the Institute for Health Metrics and Evaluation at the University of Washington’s School of Medicine in the United States of America. The results were released on 13 September.

While most people who develop COVID-19 fully recover, it is estimated that 10–20% develop mid- to long-term effects that include fatigue, breathlessness and cognitive dysfunction.

“While there is much we still need to learn about long COVID, especially how it presents in vaccinated versus unvaccinated populations and how it impacts reinfections, this data highlights the urgent need for more analysis, more investment, more support, and more solidarity with those who experience this condition,” said Hans Henri P Kluge, WHO Regional Director for Europe.

https://bit.ly/3QHqsfb

**Cover photo**

Children whose home was damaged by flooding play in a stream in Madyan, Pakistan. As a result of catastrophic flooding in the country an estimated 634 000 people have been obliged to move into camps, and 6.4 million are in urgent need of humanitarian aid.

**Figure:**
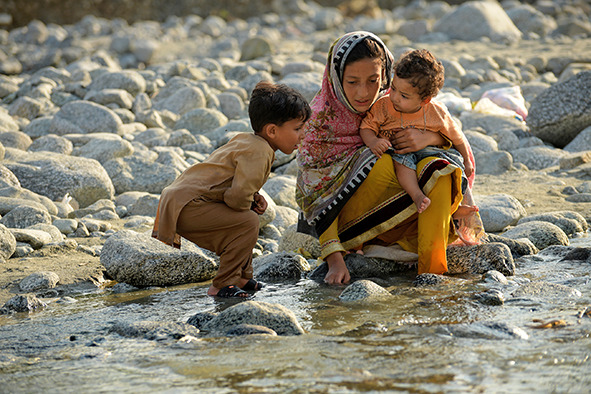
WHO/Mobeen Ansari

## Unclean health facilities

An estimated one in two health facilities worldwide lack basic hygiene products and services such as water and soap or alcohol-based hand rub.

According to the latest Joint Monitoring Programme report by WHO and the United Nations Children’s Fund published on 30 August, the estimated 3.85 billion people who use those facilities face increased risk of infection, including 688 million people who receive care at facilities with no hygiene products or services at all.

“Hygiene facilities and [hygienic] practices in health care settings are non-negotiable,” said Maria Neira, WHO Director, Department of Environment, Climate Change and Health, stressing their importance to COVID-19 pandemic preparedness, prevention and recovery.

https://bit.ly/3TKCreH

## Air quality repository launched

WHO launched an online repository of air quality management materials. The repository was launched on 7 September, World Clean Air Day, and is designed to serve as a one-stop-shop for tools and guidance documents published by United Nations agencies and international institutions related to air quality policies, monitoring methods, funding opportunities and educational programmes.

A complementary report, focused on policymaking, health impact assessment, valuation of health cost and training programmes related to air quality management was also due for release in September.

WHO data indicate that roughly 99% of the global population breathes air that exceeds WHO limits for pollutants that include fine particulate matter (2.5 microns or less) which causes cardiovascular and respiratory disease and cancers, with people in low- and middle-income countries suffering from the highest exposures.

https://bit.ly/3BdOh8E

**Looking ahead**

20–22 October, Caribbean Congress on Adolescent and Youth Health. https://bit.ly/3epuawu

16–18 November, Meeting of the Parties to the Protocol on Water and Health https://bit.ly/3erwaEo

22–24 November, The Healthy Cities Annual Business Meeting and Technical Conference. Copenhagen, Denmark. https://bit.ly/3D1mNG2

